# Use of complementary and alternative medicine (CAM) among emergency department (ED) patients in Sweden

**DOI:** 10.1186/s12906-020-03126-9

**Published:** 2020-10-31

**Authors:** Jenny M Carlsson, Madelene Vestin, Kristofer Bjerså

**Affiliations:** 1 Accident and Emergency Department, Kungälv Hospital, S-4234 Kungälv, Sweden; 2 Department of Surgery, Institute of Clinical Sciences, Sahlgrenska Academy, University of Gothenburg, S-41345 Gothenburg, Sweden

**Keywords:** Emergency service, complementary therapies, Communication, Patients, Sweden

## Abstract

**Background:**

It has been suggested that the combination of complementary and alternative medicine (CAM) with conventional medicine carries a risk of adverse effects. The prevalence of CAM usage among patients in the Swedish emergency department (ED) is unknown. Hence, the aim of this study was to investigate CAM use among visiting patients at a Swedish ED.

**Method:**

A cross-sectional descriptive study was performed between August and October 2016 at an ED in Sweden. The questionnaire included 16 items regarding CAM use, factors associated with CAM use and patient healthcare communication and was distributed to 1600 patients.

**Results:**

A total of 1029 questionnaires was returned (RR 64.3%). Current CAM use was reported by 7.9%, during the last year by 38.0%, and within lifetime by 72.9%. Factors associated with CAM use were: being a woman, middle-aged, in full-time employment, with secondary education level, higher use of non-prescription drugs and lower use of prescription drugs. Patient healthcare personnel communication about CAM was found to be approximately 5%.

**Conclusion:**

CAM usage exists among patients visiting Swedish EDs and almost one in ten uses CAM on the same day as the ED visit. CAM usage is associated with demographic factors. However, communication about CAM usage with ED personnel is poor.

**Supplementary Information:**

**Supplementary information** accompanies this paper at 10.1186/s12906-020-03126-9.

## Background

Complementary and alternative medicine (CAM) is a subject that has aroused great interest in general as well as in research during the last few decades [1–3]. A coherent definition of CAM is difficult to find due to the great variety of therapies, systems, and explanatory models available. According to the National Center for Complementary and Integrative Health (NCCIH), which is a part of the United States National Institutes of Health (NIH) and was formerly known as the National Center for Complementary and Alternative Medicine (NCCAM), complementary medicine is defined as: “a non-mainstream practice used together with conventional medicine”, and alternative medicine as: “a non-mainstream practice used in place of conventional medicine” [4]. On the other hand, what is considered conventional medicine also differs between countries due to variations in culture, traditions and laws. Definitions used in this study are chosen from a Swedish thesis on CAM in surgical care [5] in which the definitions of conventional, complementary, alternative and integrative medicine are adapted to the Swedish healthcare context (see Table 1).

**Table 1:** Definitions of conventional, complementary, alternative and integrative medicine adjusted to the Swedish context based on definitions presented by Bjerså [5]

**Alternative Medicine**	
Treatments given with the aim of curing or preventing disease, promoting or maintaining health and wellbeing, or as symptom management instead of conventional medicine.	
**Complementary and Alternative Medicine (CAM)**	
Generic term for all therapies and medical systems not included, or not perceived, as a standardised part of conventional medicine.	
**Complementary Medicine**	
Treatments given with the aim of curing or preventing disease, promoting or maintaining health and wellbeing, or for symptom management in parallel with conventional medicine.	
**Conventional Medicine**	
Treatment regulated by the current governmental, political healthcare system and given by registered healthcare professions in public hospitals, district healthcare centres, home nursing and nursing homes.	
**Integrative Medicine**	
Evidence-based treatments given in collaboration and with dialogue between conventional medicine and alternative and complementary medicine practitioners.	

International research demonstrates a prevalence of lifetime CAM use between 10 and 75% [2]. Increased usage among the general population in Western populations has been seen since the 1990s, and the levels remain similar up to the current date [2, 6]. In Europe, a large systematic literature review from 2012 focusing on the prevalence of CAM usage reports overall CAM use across European countries as between 0.3 and 86%, with a mean and median of 30% [7]. In Scandinavia, the prevalence of lifetime CAM use is indicated at the levels of 34% in Norway, 45% in Denmark and 49% in Sweden [8]. There is a lack of more recent investigations in the general Swedish population.

In terms of specific CAM therapies, herbal therapy is listed as the most frequently used CAM both in the United States, estimated at 18.6% [9], and in Europe, estimated as varying between 5.9–48.3%. A European analysis conducted in 2010–2012 reveals natural products, homeopathy, chiropractic, acupuncture and reflexology as the five most common CAM therapies in descending order [7].

Predictors of increased levels of CAM use in the general population are reported as: being female, aged 30–59 years, with a higher annual household income, perceived poorer health [3, 9, 10], and chronic conditions and/or serious illness [11–13]. Musculoskeletal problems are commonly given as reasons for CAM use and also dissatisfaction with conventional medicine and healthcare systems [7]. However, it has been observed that CAM users often tend to seek help from conventional care to a greater extent than non-users [8, 13], and also to combine treatments and prescribed drugs from conventional medical care with the use of CAM [14, 15].

The use of CAM at the same time as conventional medical treatment has been a relatively unknown field of research in Europe, but dialogues concerning evidence have been initiated [16]. Potential safety aspects concerning combined CAM-conventional medicine usage have been discussed, including risks of interactions, toxicity and altered blood coagulation time [15, 17]. Conclusions from the study of these safety aspects emphasise the need for good communication between patients and healthcare professionals about CAM usage. However, previous research indicates limited performance of such communication [5, 14].

Care provision in the hospital’s emergency department (ED) is uniquely determined by its wide variety of patients from a demographical perspective, its broad scope of medical practices in terms of assessment and management, and its unceasing patient communication. Internationally, research on CAM use combined with the emergency department context is relatively limited, with only one published systematic review based on 18 studies [18]. The prevalence of CAM use on the same day as an ED visit is reported with a range of 10–25% [18] and usage for the same reason as the medical cause of the ED visit is estimated at 10–28% [19, 20]. However, to our knowledge, no studies on CAM use and prevalence combined with ED visits in Sweden, or even in Scandinavia, have been performed. Also, variance of CAM use in different medical fields, as well as patient-health care provider communication concerning the use of CAM has not been reported on in an ED setting in previous research.

Hence, the aim of this study was to investigate CAM use among patients at a Swedish ED, with an emphasis on: (i) present and previous use of CAM, (ii) associated factors for present and previous CAM use, (iii) describing the most commonly used therapies, and (iv) describing the occurrence of communication about CAM use with healthcare personnel.

## Methods

### Design

This study was conducted with cross-sectional data collection by paper questionnaire at a Swedish mid-size ED.

### Setting and sample

The study was conducted at an ED in a middle-sized hospital in the west of Sweden. The ED provides care for patients > 16 years old in the specialities of medicine, surgery and orthopaedics, with an average visit rate of approximately 80 patients/day. Patients visiting the ED during the time of data collection were invited to participate verbally by a staff member, as well as in written forms on the front page of a questionnaire. By returning a completed questionnaire, informed consent was considered to have been given. Inclusion criteria were: sufficient skills in reading and writing Swedish, and being over 16 years old. An exclusion criterion was cognitive disability that prevented the patient from understanding the information and questions in the questionnaire.

### Data collection

Data collection was performed around the clock between 30 August and 11 October 2016. The rationale behind this ten-week time frame was to capture variations in patient flow as well as in demographical variation. Study recruitment was performed within the ordinary care by the regular staff; by medical secretaries during daytime and by nurses or nurse-assistants by night-time. Concerning number of visits during this period, a total of 3665 patients visited the ED accordioning to the hospital administration. Concerning this study, a total of 1600 patients were approached and asked to participate and provided with a paper questionnaire at arrival to the ED and collected when leaving the ED for home or a ward. The questionnaire with return envelope was distributed by the medical secretary, a registered nurse or a nurse assistant. In total, 1029 questionnaires were returned before the individual patient left the ED (response rate 64.3%).

Additionally, total patient population data for the data-collection period, such as age, sex distribution and medical speciality distribution (medicine, surgery, or orthopaedics), was retrieved from the department’s organisational system in order to perform a non-response analysis.

### The questionnaire

The content of this study’s questionnaire was set to include CAM use, communication concerning CAM use, medical information and general patient demographics. Construction of the questionnaire and its content was done with inspiration from previous international and national studies [12, 20, 21]. A first draft of the questionnaire was assessed for face validity by a postdoctoral researcher in the field of CAM, after which minor adjustments were performed.

The final version, a distributed paper questionnaire in Swedish, comprised of five pages including information about the study and its aims, the topic of CAM, research ethics, instructions for participation and study questions. In total, 16 questions/items were included in the questionnaire (Available as Additional file):

- The first item listed 20 different CAM therapies with questions about use and frequency of use.
- The remaining 15 items included factors that assessed communication about CAM between patients and healthcare staff as well as factors associated with CAM use: age, sex, marital status, education level, type of residential area, employment status, household annual income, assigned priority level of care for the ED visit (not included in the analysis); current medical discipline/specific problem or reason for visiting the ED, i.e. medicine, surgery or orthopaedics; use of prescription and non-prescription drugs; medical diagnosis; and self-reported chronic diseases.

All items had the response options of yes/no or an open response, and to be considered a CAM user, a patient indicated the use of one or several therapies.

### Data analysis

Data was compiled using IBM Statistical Package for Social Sciences® (SPSS®) version 23. Descriptive variables were calculated and presented regarding numbers of participants (*n*) and percentage (%); continuous variables were calculated and presented as mean, standard deviation (SD) and min-max. To analyse the associations between CAM use (today and lifetime) and sex, marital status, employment status, medical areas and self-reported chronic disease, the Pearson’s Chi^2^ test was used. The Pearson’s Chi^2^ test was also used in the analysis of CAM communication and sex. The association between CAM use (today and lifetime) and participant age was analysed using the independent student’s t-test. The Mann-Whitney *U* test was used to analyse the association between CAM use (today and lifetime) and number of prescription or non-prescription drugs used, household annual income, level of education and residential area. A non-response analysis was performed to assess the difference between the study participants and the population in the areas of sex and medical speciality. The level of statistical significance was set at *p* < 0.05.

### Ethical considerations

Approval for conducting this study was given by the Head of Department at the Accident and Emergency Department, Kungälv Hospital. All participants received written information about the aim of the study, about the principles for voluntary participation and that participation or relinquished involvement would not affect their own current or future care. This study was performed in accordance with the ethical research approach stated in the Helsinki Declaration principles regarding research on human subjects [22], and all data was treated confidentially and with full disclosure. This study was performed as part of a master’s thesis project within advanced nursing science at the University of Linköping, Sweden. Hence, it was conducted in accordance with regulations in the Swedish Ethical Review Act SFS 2003:406; Prop. 2007/08:44, approved, monitored and reviewed by the University of Linköping and thus not in need of undergoing further ethical reviewing.

## Results

A total of 1029 questionnaires were returned, resulting in a response rate of 64.3%. Participants’ demographics are presented in Table 2.

**Table 2:** Participant demographics

		Mean	SD	%	***n=***	Min-Max
**Sex (***n*_*total*_ = 998)	Male/Female			49.1/50.9	490/508	
**Age (***n*_*total*_ = 996)	Total	53.6	19.7			16–95
	Male	53.9			487	16–94
	Female	53.3			505	16–95
**Marital status (***n*_*total*_ = 980)	Married			48.5	485	
	Partner			20.6	202	
	Living apart			6.0	59	
	Single			13.0	133	
	Separated			5.0	49	
	Widow/er			6.3	62	
**Highest level of education (***n*_*total*_ = 978)	Elementary school			2.5	29	
	Secondary education			47.8	467	
	Folk/Public college			3.8	37	
	College/University			27.9	273	
**Residential area (**n_*total*_ = 997)	Urban area > 25,000 inhabitants			34.2	334	
	Small town < 10,000 inhabitants			26.0	254	
	Village < 500 inhabitants			15.3	149	
	Countryside			24.6	240	
**Employment status (***n*_*total*_ = 977)	Full-time employment			45.5	445	
	Part-time employment			10.3	101	
	Unemployed			2.0	20	
	Student			4.1	40	
	Sick leave			3.5	34	
	Pension			34.5	337	
**Household annual income** thousand Swedish krona, SEK *(n*_*total*_ = 923)	< 100,000			6.0	55	
	100–300,000			29.4	271	
	300–700,000			46.2	426	
	700,000–1 million			14.0	129	
	> 1 million			4.6	42	
**Medical areas (***n*_*total*_ = 982)	Medicine			41.6	409	
	Surgery			26.5	206	
	Orthopaedics			31.9	313	
**Number of prescription drugs (***n*_*total*_ = 1029)		2.15				0–20
**Number of non-prescription drugs** *(n*_*total*_ = 1029)		.47				0–8
**Medical diagnosis (***n*_*total*_ = 1029)	Cardiovascular			14.3	147	
	Cerebrovascular			3.0	31	
	Hypertension				24.8	255
	Kidney				1.6	16
	Liver				.9	9
	Pulmonary				3.4	35
	Cancer				4.6	47
	Diabetes				6.6	68
**Self-reported chronic disease (***n*_*total*_ = 957)					27.7	265
**Received questions about CAM usage from healthcare provider (***n*_*total*_ = 942)				4.2	40	
**Self-reported CAM usage to healthcare provider (***n*_*total*_ = 797)				5.0	40	

A non-response analysis for comparison between the study participants and the total population was conducted, based on retrieved data of the patient population during the data-collection period. There was no significant difference between sex distribution (*p* = 0.569), but a significant difference was found in the distribution of medical area (*p* = 0.020), with a higher number of orthopaedic visits and fewer medicine visits in the study sample compared with the total population. The distribution of surgical visits was equal between the groups.

### CAM usage and factors associated with CAM usage

The results indicate that 7.9% (*n*_*total*_ = 1029) of the participants used CAM on the same day as they visited the ED, and the number of non-prescription drugs regularly used was found to be associated with CAM use today, see Table 3. Nearly twice as much non-prescription drug consumption was observed among participants with current CAM use compared to non-CAM users (*p* = < 0.001; *n* = 1029; mean 0.85 vs. 0.44).

**Table 3:** Demographic factors associated with CAM use

CAM associates	CAM use today *p*-value	CAM use 12 months *p*-value	CAM use lifetime *p*-value
Number of non-prescription drugs^1^ (*n* = 1029)	< 0.001	< 0.001	< 0.001
Highest level of education^1^ (*n* = 978)	0.005	< 0.001	< 0.001
Employment status^2^ (*n* = 977)	0.136	< 0.001	< 0.001
Self-reported chronic disease^2^ (*n* = 957)	0.373	0.018	0.849
Medical area^2^ (*n* = 982)	0.463	0.755	0.824
Sex^2^ (*n* = 998)	0.498	< 0.001	< 0.001
Number of prescription drugs^1^ (*n* = 1029)	0.793	< 0.001	0.009
Residential area^1^ (*n* = 977)	0.841	0.819	0.428
Marital status^2^ (*n* = 980)	0.853	0.539	0.058
Household annual income^1^ (*n* = 923)	0.858	< 0.001	< 0.001
Age^3^ (*n* = 996)	0.994	0.055	< 0.001

^1^ Mann-Whitney *U*

^2^ Pearson’s Chi^2^

^3^ Independent student’s t-test

Concerning CAM use during the last 12 months, 38.0% (n_*total*_ = 1029) of the participants stated use of CAM. CAM use during the last year was statistically associated with sex, level of education, household annual income, employment status, self-reported perception of suffering from a chronic disease, as well as number of non-prescription drugs and number of prescription drugs (see Table 3); There were significantly more women than men using CAM during the last 12 months (60.4% vs. 39.6%; n_*total*_998; *p* < 0.001). This usage was also associated with higher education; secondary education (*p* < 0.001;n_*total*_ = 978), i.e. education after primary school, was more common within the CAM use group (88.7%;*n* = 337) compared to non-CAM (73.6%;*n* = 440). Annual household income was higher among the CAM users, and employment status differed; CAM users worked full-time in higher extent (55.8% vs. 39.1%) and less of the CAM users were retired (20.4% vs. 43.4%), compared to the non-CAM users (*p* < 0.001; *n* = 977). Self-reported chronic disease was found as significantly different in distribution (*p* = 0.018; *n* = 957) between users and non-users of CAM during the last 12 months; 23.3% of the CAM users compared to 30.4% among the non-CAM users. The number of non-prescription drugs regularly used was higher among CAM users (mean 0.63 vs. 0.38) but the use of prescription drugs was lower (mean 1.69 vs. 2.42) compared to non-CAM users during the last 12 months (*p* < 0.001; n_non-prescription_ = 1029/ n_prescription_ = 1029).

Use of CAM within the lifetime was reported by 72.9% (*n*_*total*_ = 1029). We found that sex, age, level of education, household annual income, employment status, number of non-prescription drugs and number of prescription drugs were associated with lifetime CAM usage (Table 3). Lifetime CAM use was significantly more common among women than men (55% vs. 45%; *p* < 0.001) and with an overall lower mean age (52 vs. 58; *p* < 0.001). Lifetime CAM use was most common within the group of participants who reported secondary education as their highest level of education (84.4% vs 71.8%; *n*_*total*_ = 978), as well as annual household income was higher among the CAM users. Concerning employment status (*n* = 977; *p* < 0.001), full-time employee was most common among lifetime CAM users (49.6% vs. 39.3%), number of students lower (2.5% vs. 6.5%) as well as umber of retired participants (30.4% vs. 40.8%) compared to the non-CAM users. The number of non-prescription drugs regularly used was higher among those who listed lifetime CAM use (mean 0.52 vs. 0.34) but the use of prescription drugs was lower (mean 1.99 vs. 2.57).

### Usage within CAM-specific therapies

The results demonstrate variations in CAM therapy usage, whereby some therapies appeared more frequently than others (see Table 4).

**Table 4:** Therapy specified CAM use

CAM therapy	CAM use today^a^	CAM use last 12 months^a^	CAM use lifetime^a^
Ayurveda (*n* = 43)	0.1%	0.7%	3.3%
Homeopathy (*n* = 140)	0.0%	1.3%	12.3%
Psychotherapy, Cognitive Behavioural Therapy (CBT) (*n* = 220)	0.5%	4.0%	16.5%
Meditation, Mindfulness, etc.(*n* = 245)	1.6%	5.6%	15.3%
Healing, Reiki, etc. (*n* = 93)	0.3%	2.1%	6.4%
Yoga (*n* = 330)	0.9%	8.9%	21.5%
Chiropractic (*n* = 407)	0.4%	8.2%	30.8%
T’ai Chi, Qi Gong (*n* = 102)	0.1%	1.5%	8.4%
Acupuncture, Acupressure (*n* = 398)	0.7%	6.2%	31.2%
Massage, Shiatzu, Tactile massage (*n* = 610)	0.6%	17.3%	40.8%
Zone therapy, Reflexology (*n* = 115)	0.2%	1.6%	9.3%
Naprapathy (*n* = 279)	0.2%	5.5%	21.2%
Herbal medicine (*n* = 219)	1.1%	4.6%	14.5%
Bowen therapy (*n* = 11)	0.0%	0.1%	1.0%
Iridology (*n* = 34)	0.0%	0.1%	3.2%
Osteopathy (*n* = 153)	0.1%	3.5%	11.2%
Kinesiology (*n* = 52)	0.0%	1.2%	3.9%
Sense therapies (i.e. light-, music-, aroma-therapy) (*n* = 52)	0.4%	0.8%	3.5%
Rosen method (*n* = 18)	0.0%	0.2%	1.6%
Health foods (*n* = 571)	4.5%	13.6%	33.3%

^a^ Specified percentages are calculated on *n* = 1029. Each participant had the opportunity to indicate use of one or several different therapies, both in CAM use today and CAM use lifetime

Use of herbal medicine, the third most commonly used therapy on the same day as the ED visit, was significantly different (*p* = 0.047; *n* = 982) in patient distribution between the medical areas of the ED, i.e., medicine, surgery and orthopaedics. Of participants who reported usage of herbal medicine, 72.7% had a reason for the ED visit listed in the medicine speciality and 27.3% in surgery. No significant difference was found between medical area and health foods (*p* = 0.248; *n* = 982) or between medical area and meditation/mindfulness (*p* = 0.886; *n* = 982), which were the first and second most frequently used therapies on the same day as the ED visit. Concerning CAM use during the last 12 months, no significant different in medical area was found among massage therapies (*p* = 0.945: *n* = 981) and health foods (*p* = 0.930: *n* = 981) users. However, a statistical difference was found concerning yoga practice (*p* = 0.014: *n* = 981); significantly more patient using yoga during the last 12 months visited the ED for orthopaedic reasons (46.6% vs. 30.3%) compared to non-yoga users. Furthermore, no significant difference was observed between medical area and the most frequently used therapies within lifetime: massage methods (*p* = 0.772; *n* = 982), health foods (*p* = 0.205; *n* = 982), acupuncture/acupressure (*p* = 0.825; *n* = 982), and chiropractic (*p* = 0.325; *n* = 982).

### Communication about CAM

Irrespective of CAM use, only 4.2% of the participants (*n*_*total*_ = 947) responded that they were asked questions about CAM usage by healthcare professionals during their ED visit. Furthermore, only 5.1% of all participants and 13.0% of patient using CAM at the same day as the ED visit (n_*total*_ = 799) reported that they informed the healthcare professionals at the ED about their none or current CAM use. No significant sex difference was found in communication about CAM, either among personnel asking about CAM use (*p* = 0.627; *n* = 920) or among patient self-reporting CAM use (*p* = 0.740; *n* = 778).

## Discussion

According to the results of this unique study, the use of CAM exists among patients in the Swedish ED setting. It is possible that approximately 8% of patients in Sweden use CAM on the same day as they visit the ED, just below 40% during the last year, and 73% of ED patients use CAM within a lifetime.

Our findings of higher CAM use among women and middle-aged people are consistent with several previous studies [3, 6, 15]. Clarke and McLachan [23] found that self-medication for menopausal symptoms was indicated by many women, but other reasons for CAM use were also reported, such as self-care and the prevention of medical problems. Nearly half of all CAM users in this study (46.2%; *n* = 426) listed their household annual income as SEK 300–700,000 (approx. €31,400–73,300). Based on Swedish annual income levels by mean [24], this is considered to be within a middle-class income range. Secondary education as the highest educational level was also significant for CAM use in this study. The results regarding both income and education levels in our study may differ from previous findings. In a systematic review by Frass et al. [6], the authors found that a higher income level was a predictor for increased CAM use in 11 of 16 publications. Frass et al. [6] and Kristoffersen et al. [11] also report that CAM use was more likely among individuals with a university education. By combining their results of higher income and educational levels, we can see that there is an indication of more prevalent CAM use within higher socio-economic classes. Thus, our findings indicate that prevalent CAM use within the middle socio-economic class also applies to Sweden.

In terms of specific CAM therapies, this study reveals that massage methods, health foods, acupuncture/acupressure and chiropractic are the most commonly used within a lifetime in a Swedish population sample. The reported preferences for specific CAM therapies vary between countries depending on traditions and healthcare systems [6]. Nevertheless, a study by Eklöf and Tegern [25] found massage, acupuncture and chiropractic to be the most frequently used therapies within Sweden at the beginning of the millennium. Our results thus show a continued interest in Sweden in these three specific therapies. Eklöf and Tegern [25] also presented herbal remedies as commonly used in Sweden. The finding of health foods as the second most commonly used therapy in this study cannot be equated with such herbal remedies, but may be seen as a continued interest in the same therapeutic area. The estimated lifetime CAM use by almost three-quarters of all patients found in this study corresponds to previous levels estimated within the general Western population [7]. Hence, our study supports previous findings of high usage levels and preferences concerning CAM among the general population and indicates continued interest, even within the Swedish population.

High levels of CAM usage by patients emphasises the importance of knowledge and communication about CAM within conventional healthcare. Only about 5% indicated communication about CAM with the ED personnel in this study. This lack of CAM communication is also reported in previous national and international research [5, 26–28]. Why such communication is limited within conventional healthcare is unknown. Wardle and Adams [29] found that patients did not communicate about CAM due to fear of disclosure in case of a negative response from conventional healthcare providers. Expected disapproval of CAM use was also given as a reason for this non-communication by Jong et al. [30], but they also found a desire for communication about CAM among participants in their study. In a previous national study within the surgical context at Sweden’s university hospitals, perceived knowledge about CAM was valued by 95.7% of the participating nurses, surgeons and physical therapists as minor or no knowledge at all [21]. Furthermore, Waterbrook et al. [20] found that both physicians and patients at the ED expressed the importance of being educated about CAM. This was also found in a previous publication from our research group [28] as three-fifths of ED staff expressed a wish to gain more knowledge in the field of CAM. Based on these findings, limited communication about CAM may be understood as partly a fear of disclosure and partly a lack of knowledge about the topic of CAM. Based on this, we recommend more educational interventions about CAM within conventional healthcare.

From the perspective of previously presented non-communication about CAM, the current CAM use by almost one in ten ED patients (7.9%) that we found is most likely present without the awareness of treating physicians and nurses, and must be considered a potential patient safety risk. Acute life-threatening situations, which sometimes present in an ED, may lead to a marked variability in drug pharmacokinetics and toxicity [23] for example, herbal CAM therapies have been observed to cause increased bleeding [18, 31]. It is further likely, but at present unknown, that some patients seek care at the ED due to reactions or adverse effects following CAM treatments or therapies. Non-awareness and a lack of knowledge about CAM use in an ED setting may cause serous patient harm. In summary, our results have uncovered risks of decreased opportunities for ED healthcare professionals to carry out patient-safe care as incumbent upon them by Swedish law.

### Limitations

Some limitations within this study need to be reflected upon. Since no previous comparative study on the Swedish ED context has been performed and the study aim was to be identified, the questionnaire was self-developed using previous knowledge, adjusted to the study aim and applicability. No pre-test of the questionnaire was performed, which may be seen as a weakness and a threat to the validity and reliability of the study as this, for example, decreases the assurance of appropriate measurements. However, the content of the questionnaire was assessed by a postdoctoral researcher in the field of CAM, which strengthens the face validity.

During the data collection, our research team became aware of a specific employment status that may be present in the Swedish population: “parental leave”. This questionnaire lacks this as an answering alternative, which may have affected the results. In Sweden, parental leave is a part of the social insurance system and offered for 480 days per child up to the year when the child becomes eight or 12 years old. Furthermore, despite instructions to the participants regarding the “assigned priority level of care for the emergency department visit”, several participants misinterpreted this and left the question unanswered. For this reason, this question was excluded from the analysis.

Except for the exclusion criteria, a large number of patients were not invited to participate in the study due to questionnaires distribution made by the regular personnel. During daytime, study recruitment was mainly handled by the medical secretaries. However, during night time some patients was not reached with study invitation due to the absence of the medical secretaries and only nurses and nurse assistants handled this task. The main reason for missed out patient recruitment by the nurses and nurse assistants was their need to perform more prioritized tasks. For the same reason, the number of patient excluded and missed during data collection period was not registered. This fact reduced the study population and must be considered as a weakness.

The response rate for this study was 64.3%, which may be seen as a threat to the results. However, in the context of the study setting and the sample of acutely ill or injured patients, often experiencing stress and anxiety, it can be considered a relatively high level of response. The response rate of 64.3% and a sample size of 1029 also strengthen the results of this study as a response rate of ≥60% and a sample size of ≥1000 are considered criteria for quality assessment in CAM research [2]. In addition to this, the non-response analysis indicated that the sample is adequately representative of the total population in all comparable variables except medical area.

## Conclusion

CAM therapies are frequently used by patients visiting the ED in Sweden, and almost one in ten uses CAM on the same day as the ED visit. Increased CAM usage was associated with sex, age, and socioeconomical factors, consistent with previous international research. CAM use was also associated with lower use of prescription drugs and higher use of non-prescription drugs. A lack of communication about CAM use between the ED healthcare professionals and patients was evident.

## Supplementary Information

**Additional file 1.**

## Data Availability

Data will be available upon request.

## Abbreviations

CAM: Complementary and alternative medicine
ED: Emergency department
NCCAM: The national center for complementary and alternative medicine
NCCIH: The national center for complementary and integrative health
NIH: The United States national institutes of health

## References

[1] Eisenberg DM, Davis RB, Ettner SL, Appel S, Wilkey S, Van Rompay M (1998). Trends in alternative medicine use in the United States, 1990-1997: results of a follow-up national survey. JAMA.

[2] Harris P, Cooper K, Relton C, Thomas K (2012). Prevalence of complementary and alternative medicine (CAM) use by the general population: a systematic review and update. Int J Clin Pract.

[3] Kristoffersen AE, Stub T, Salamonsen A, Musial F, Hamberg K (2014). Gender differences in prevalence and associations for use of CAM in a large population study. BMC Complement Altern Med.

[4] The National Center for Complementary and Integrative Health (NCCIH). Complementary, Alternative, or Integrative Health: What’s In a Name? USA: National Institutes of Health (NIH); [updated 2016; cited 2018 13 April]. Available from: https://nccih.nih.gov/health/integrative-health.

[5] Bjerså K (2012). Complementary and alternative therapies in surgical care.

[6] Frass M, Strassl RP, Friehs H, Müllner M, Kundi M, Kaye AD (2012). Use and acceptance of complementary and alternative medicine among the general population and medical personnel: a systematic review. Ochsner J.

[7] Eardley S, Bishop FL, Prescott P, Cardini F, Brinkhaus B, Santos-Rey K (2012). CAM use in Europe–The patients’ perspective. Part I: A systematic literature review of CAM prevalence in the EU.

[8] Hanssen B, Grimsgaard S, Launsø L, Fønnebø V, Falkenberg T, Rasmussen NK (2005). Use of complementary and alternative medicine in the Scandinavian countries. Scand J Prim Health Care.

[9] Tindle HA, Davis RB, Phillips RS, Eisenberg DM (2005). Trends in use of complementary and alternative medicine by US adults: 1997-2002. Altern Ther Health Med.

[10] Nilsson M, Trehn G, Asplund K (2001). Use of complementary and alternative medicine remedies in Sweden. A population-based longitudinal study within the northern Sweden MONICA project. J Intern Med.

[11] Kristoffersen AE, Norheim AJ, Fønnebø VM. Complementary and alternative medicine use among Norwegian cancer survivors: gender-specific prevalence and associations for use. Evid Based Complement Altern Med. 2013;Article ID 318781:10. 10.1155/2013/318781 PMC3625602 23606877

[12] Oxelmark L, Lindberg A, Löfberg R, Sternby B, Eriksson A, Almer S (2016). Use of complementary and alternative medicine in Swedish patients with inflammatory bowel disease: a controlled study. Eur J Gastroenterol Hepatol.

[13] Steinsbekk A, Adams J, Sibbritt D, Jacobsen G, Johnsen R (2007). The profiles of adults who consult alternative health practitioners and/or general practitioners. Scand J Prim Health Care.

[14] Hunt KJ, Coelho HF, Wider B, Perry R, Hung S, Terry R (2010). Complementary and alternative medicine use in England: results from a national survey. Int J Clin Pract.

[15] Taylor DM, Walsham N, Taylor SE, Wong L (2004). Use and toxicity of complementary and alternative medicines among emergency department patients. Emerg Med Australas.

[16] Fischer FH, Lewith G, Witt CM, Linde K, von Ammon K, Cardini F (2014). High prevalence but limited evidence in complementary and alternative medicine: guidelines for future research. BMC Complement Altern Med.

[17] Taylor DM, Walsham N, Taylor SE, Wong L (2006). Potential interactions between prescription drugs and complementary and alternative medicines among patients in the emergency department. Pharmacotherapy J Hum Pharmacol Drug Ther.

[18] Jatau AI, Aung MMT, Kamauzaman THT, Chedi BA, Sha’aban A, Ab Rahman AF (2016). Use and toxicity of complementary and alternative medicines among patients visiting emergency department: systematic review. J Intercultural Ethnopharmacol.

[19] Allen R, Cushman LF, Morris S, Feldman J, Wade C, McMahon D (2000). Use of complementary and alternative medicine among Dominican emergency department patients. Am J Emerg Med.

[20] Waterbrook AL, Southall JC, Strout TD, Baumann MR (2010). The knowledge and usage of complementary and alternative medicine by emergency department patients and physicians. J Emerg Med.

[21] Bjerså K, Victorin ES, Olsén MF (2012). Knowledge about complementary, alternative and integrative medicine (CAM) among registered health care providers in Swedish surgical care: a national survey among university hospitals. BMC Complement Altern Med.

[22] World Medical Association (2013). Declaration of Helsinki: ethical principles for medical research involving human subjects. JAMA.

[23] Clarke SJ, McLachlan AJ (2011). Interaction between complementary and alternate medicine with conventional anti-cancer medicine. CancerForum.

[24] Statistics Sweden (SCB) (2018). Total income from employment and business 2016.

[25] Stockholms läns landsting (2001). Stockholmare och den komplementära medicinen : befolkningsstudie angående inställning till och användning av komplementär medicin genomförd under år 2000 i Stockholms läns landsting.

[26] Gulla J, Singer AJ (2000). Use of alternative therapies among emergency department patients. Ann Emerg Med.

[27] MacLennan AH, Myers SP, Taylor AW (2006). The continuing use of complementary and alternative medicine in South Australia: costs and beliefs in 2004. Med J Aust.

[28] Vestin M, Carlsson JM, Bjerså K (2018). Emergency department staffs’ knowledge, attitude and patient communication about complementary and alternative medicine–a Swedish survey. Eur J Integr Med.

[29] Wardle JJL, Adams J (2014). Indirect and non-health risks associated with complementary and alternative medicine use: an integrative review. Eur J Integr Med.

[30] Jong MC, van de Vijver L, Busch M, Fritsma J, Seldenrijk R (2012). Integration of complementary and alternative medicine in primary care: what do patients want?. Patient Educ Couns.

[31] Posadzki P, Watson L, Ernst E (2013). Herb–drug interactions: an overview of systematic reviews. Br J Clin Pharmacol.

