# Supplementary material for: Use of complementary and alternative medicine (CAM) among emergency department (ED) patients in Sweden
Source: BMC Complement Med Ther. 2020 Oct 31;20:327. doi: 10.1186/s12906-020-03126-9 (PMC7603685; doi:10.1186/s12906-020-03126-9)
Supplement: Supplementary file 1 — Additional file 1. [file 12906_2020_3126_MOESM1_ESM.zip › Questionnaire EnglishR4.pdf]

¤ ¤ ¤ USE of **CAM** therapies ¤ ¤ ¤

1. Different therapies are listed below. Are you currently using, or have you used one or several of these therapies? If so, when? Mark with an X in the circles. Possible to respond none to all.

	**Today**	**Sometime during the last 12 months**	**Sometime in life**
AYURVEDA	o	o	o
HOMEOPATHY	o	o	o
PSYKOTHERAPY CBT	o	o	o
MEDITATION, MINDFULLNESS, ETC	o	o	o
HEALING, REIKI, ETC.	o	o	o
YOGA	o	o	o
CHIROPRACTICS	o	o	o
TAI CHI QI GONG	o	o	o
AKUPUNCTURE AKUPRESSURE	o	o	o
MASSAGE, SHIATSU TACTILE MASSAGE	o	o	o
ZONE THERAPY/ REFLEXOLOGY	o	o	o
NAPRAPATHY	o	o	o
HERBAL MEDICINE	o	o	o
BOWEN THERAPY	o	o	o
IRIDOLOGY	o	o	o
OSTEOPATHY	o	o	o
KINESIOLOGY	o	o	o
SENSE THERAPIES e.g. light therapy, music therapy, aroma therapy	o	o	o
ROSEN METHOD	o	o	o
HEALTH FOODS i.e. prepared or healthy food, nutritional supplements or diets marketed as promoting health	o	o	o

¤ ¤ ¤ Dialog with your physician or nurse about CAM use ¤ ¤ ¤

2. During your visit to the Emergency Department today, has any physician or nurse asked you about your use of CAM therapies?

   ❑ YES ❑ NO

3. If you are currently using any CAM therapy, have you told a physician or nurse about this use during this visit at the Emergency Department?

   ❑ YES ❑ NO

¤ ¤ ¤ Medical information ¤ ¤ ¤

4. What is the reason for your visit to the Emergency Department today? (Describe with only single words you main complain, for example chest pain, stomach ache, head ache, foot injury, vertigo, etc.)

   ____

5. How many **prescribed** medications do you use regularly?

   ____

6. How many **non-prescriptive** medications do you use regularly?

   __________________s

7. Are you previously diagnosed with any of these diseases?
   - ❑ Cardiovascular disease
   - ❑ Cerebrovascular disease (Stoke/TCI)
   - ❑ High blood pressure / Hypertension
   - ❑ Kidney diseases
   - ❑ Liver diseases
   - ❑ Lung diseases
   - ❑ Malignancy (Cancer)
   - ❑ Diabetes

8. Do you perceive yourself of having a chronic disease?

   ❑ YES ❑ NO

9. After the initial triage at your visit to the Emergency Department today, which priority / colour marking did the triage nurse categorize you as?
   - ❑ Red
   - ❑ Orange
   - ❑ Yellow
   - ❑ Green

¤ ¤ ¤ General questions ¤ ¤ ¤

10. Your year of birth: ___________

11. Gender: ❑ Man ❑ Women ❑ Other

12. Marital status:
    - ❑ Married
    - ❑ Living with someone
    - ❑ In a relation but live separately
    - ❑ Single
    - ❑ Divorced / Separated
    - ❑ Widow / Widower

13. Which is your highest level of education?
    - ❑ Primary school
    - ❑ High school / vocational training education
    - ❑ Folk school / Folk collage
    - ❑ Collage / University

14. What type of area do you live in?
    - ❑ City or urban
    - ❑ Small town
    - ❑ Village
    - ❑ Countryside

A city has more than 25 000 inhabitants,
a small town less than 10 000 inhabitants,
a village less than 500 inhabitants

15. Employment
    - ❑ Working, full time
    - ❑ Working, part time
    - ❑ job seeker
    - ❑ Studying
    - ❑ Sick leave
    - ❑ Retired / Pensioner

16. What is your house holds total annual income prior to tax (including income support)?

- ❑ Below 100.000
- ❑ 100.000 – 300.000
- ❑ 300.000 – 700.000
- ❑ 700.000 – 1.000.000
- ❑ Above 1 million

THANK YOU FOR TAKING THE TIME TO ANSWER THIS QUESTIONNAIRE!
