# Supplementary material for: Use of complementary and alternative medicine (CAM) among emergency department (ED) patients in Sweden
Source: BMC Complement Med Ther. 2020 Oct 31;20:327. doi: 10.1186/s12906-020-03126-9 (PMC7603685; doi:10.1186/s12906-020-03126-9)
Supplement: Supplementary file 1 — Additional file 1. [file 12906_2020_3126_MOESM1_ESM.zip › Questionnaire SwedishR4.pdf]

¤ ¤ ¤ **KAM-användning** ¤ ¤ ¤

1. Nedan finns olika KAM-behandlingsformer listade. Har du använt dig av någon/några av dessa, och i så fall när? (Sätt ett kryss i ringarna för att markera ditt svar)

	**Idag**	**Senaste 12 mån**	**Någon gång i livet**
AYURVEDA	o	o	o
HOMEOPATI	o	o	o
PSYKOTERAPI, KBT	o	o	o
MEDITATION, MINDFULLNESS, ETC	o	o	o
HEALING, REIKI, ETC.	o	o	o
YOGA	o	o	o
KIROPRAKTIK	o	o	o
TAI CHI QI GONG	o	o	o
AKUPUNKTUR AKUPRESSUR	o	o	o
MASSAGE, SHIATSU TAKTILMASSAGE	o	o	o
ZONTERAPI, REFLEXOLOGI	o	o	o
NAPRAPATI	o	o	o
ÖRTMEDICIN	o	o	o
BOWEN TERAPI	o	o	o
IRISDIAGNOSTIK	o	o	o
OSTEOPATI	o	o	o
KINESIOLOGI	o	o	o
SINNESTERAPIE t.ex. ljusterapi, musikterapi, aromterapi	o	o	o
ROSENMETODEN	o	o	o
HÄLSOKOST	o	o	o

¤ ¤ ¤ Dialog om KAM-användning ¤ ¤ ¤

2. Har någon läkare eller sjuksköterska på Akutmottagningen ***frågat dig idag*** om du använder eller använt någon form av KAM?

   ❑ Ja ❑ Nej

3. Om Du använt KAM behandling, har ***du idag berättat*** för någon läkare eller sjuksköterska på Akutmottagningen om ditt användande?

   ❑ Ja ❑ Nej

¤ ¤ ¤ Medicinska frågor ¤ ¤ ¤

4. Vad sökte du för på akutmottagningen idag? (ange endast med enstaka ord vilket besvär, ex. bröstsmärta, buksmärta, huvudvärk, skadad fot, yrsel osv.)

   ____

5. Hur många *receptbelagda* läkemedel använder du regelbundet?

   _________________st

6. Hur många *receptfria* läkemedel använder du regelbundet?

   _________________st

7. Är du diagnostiserad med någon/några av följande sjukdom/ar?
   - ❑ Hjärt-/kärlsjukdom
   - ❑ Cerebrovaskulär sjukdom (Stoke/TIA)
   - ❑ Högt blodtryck
   - ❑ Njursjukdom
   - ❑ Leversjukdom
   - ❑ Lungsjukdom
   - ❑ Malignitet (Cancer)
   - ❑ Diabetes

8. Anser du dig ha en kronisk sjukdom?

   ❑ Ja ❑ Nej

9. Vilken prioriteringsgrad/färg har sjuksköterskan tilldelat dig idag? (informationen finns på sid. 4 i informationsbroschyren du fått)
   - ❑ Röd
   - ❑ Orange
   - ❑ Gul
   - ❑ Grön

⌑ ⌑ ⌑ **Allmänna frågor** ⌑ ⌑ ⌑

10. Födelse år_______

11. ❑ Man ❑ Kvinna

12. Civilstånd:
    - ❑ Gift
    - ❑ Sambo
    - ❑ Särbo
    - ❑ Ensamstående
    - ❑ Skild/Separerad
    - ❑ Änka/Änkling

13. Vilken är den högsta utbildningen Du har?
    - ❑ Grundskola/Folkskola
    - ❑ Gymnasium/KvalificeradYrkesskola
    - ❑ Folkhögskola
    - ❑ Högskola/Universitet

14. I vilket slags område bor Du?
    - ❑ Tätort
    - ❑ Småort
    - ❑ Samhälle
    - ❑ Landsbygd

I en tätort bor fler än 25 000 invånare, i en småort bor mindre än 10 000 invånare,
I ett samhälle bor mindre än 500 invånare.

15. Sysselsättning
    - ❑ Arbetar heltid
    - ❑ Arbetar deltid
    - ❑ Arbetssökande
    - ❑ Studerande
    - ❑ Sjukskriven
    - ❑ Pensionär

16. Vilken är **hushållets** årsinkomst före skatt, (*ev. bidrag inkluderade)*?

- ❑ under 100.000
- ❑ 100.000 – 300.000
- ❑ 300.000 – 700.000
- ❑ 700.000 – 1 miljon
- ❑ över 1 miljon

TACK FÖR ATT DU TOG DIG TID ATT BESVARA DENNA ENKÄT!
